# Supplementary material for: The causal relationship between the human gut microbiota and pyogenic arthritis: a Mendelian randomization study
Source: Front Cell Infect Microbiol. 2024 Nov 26;14:1452480. doi: 10.3389/fcimb.2024.1452480 (PMC11629706; doi:10.3389/fcimb.2024.1452480)
Supplement: Supplementary file 1 [file DataSheet1.zip › mendelian test/ebi-a-GCST90027853.csv_leave_one_out.pdf]

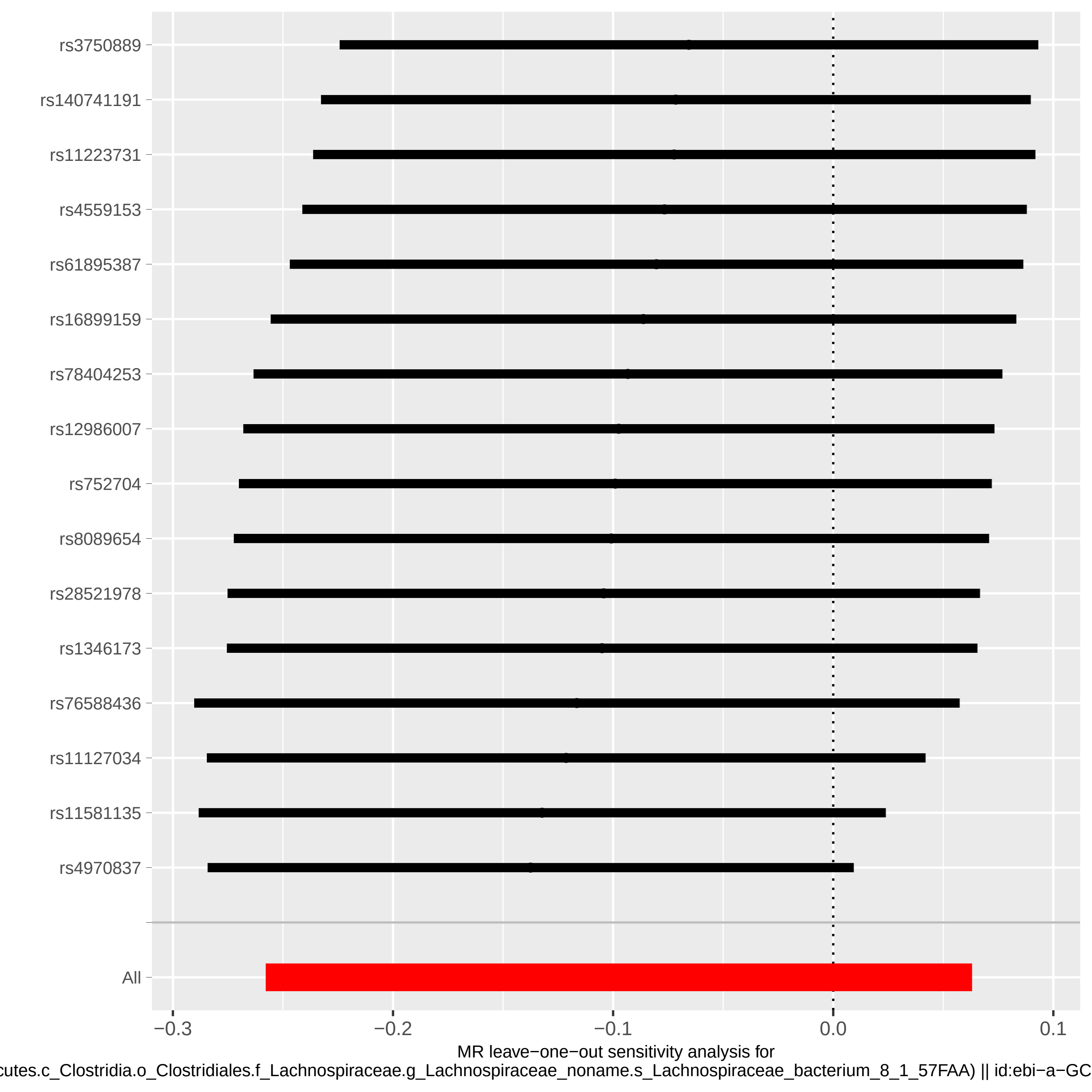

rs3750889
rs140741191
rs11223731
rs4559153
rs61895387
rs16899159
rs78404253
rs12986007
rs752704
rs8089654
rs28521978
rs1346173
rs76588436
rs11127034
rs11581135
rs4970837
All
−0.3
−0.2
−0.1
0.0
0.1
MR leave−one−out sensitivity analysis for
cutes.c_Clostridia.o_Clostridiales.f_Lachnospiraceae.g_Lachnospiraceae_noname.s_Lachnospiraceae_bacterium_8_1_57FAA) || id:ebi−a−GC
